# Emergence and evolution of yeast prion and prion-like proteins

**DOI:** 10.1186/s12862-016-0594-3

**Published:** 2016-01-25

**Authors:** Lu An, David Fitzpatrick, Paul M. Harrison

**Affiliations:** Department of Biology, McGill University, Montreal, QC Canada; Bioinformatics and Molecular Evolution Unit, NUI Maynooth, Maynooth, Ireland

**Keywords:** Prion, Evolution, Bias, Composition, Bioinformatics, Disease, Mutation, Yeast, Fungi

## Abstract

**Background:**

Prions are transmissible, propagating alternative states of proteins, and are usually made from the fibrillar, beta-sheet-rich assemblies termed amyloid. Prions in the budding yeast *Saccharomyces cerevisiae* propagate heritable phenotypes, uncover hidden genetic variation, function in large-scale gene regulation, and can act like diseases. Almost all these amyloid prions have asparagine/glutamine-rich (N/Q–rich) domains. Other proteins, that we term here ‘prionogenic amyloid formers’ (PAFs), have been shown to form amyloid in vivo, and to have N/Q-rich domains that can propagate heritable states in yeast cells. Also, there are >200 other *S.cerevisiae* proteins with prion-like N/Q-rich sequence composition. Furthermore, human proteins with such N/Q-rich composition have been linked to the pathomechanisms of neurodegenerative amyloid diseases.

**Results:**

Here, we exploit the increasing abundance of complete fungal genomes to examine the ancestry of prions/PAFs and other N/Q-rich proteins across the fungal kingdom. We find distinct evolutionary behavior for Q-rich and N-rich prions/PAFs; those of ancient ancestry (outside the budding yeasts, *Saccharomycetes*) are Q-rich, whereas N-rich cases arose early in *Saccharomycetes* evolution. This emergence of N-rich prion/PAFs is linked to a large-scale emergence of N-rich proteins during *Saccharomycetes* evolution, with *Saccharomycetes* showing a distinctive trend for population sizes of prion-like proteins that sets them apart from all the other fungi. Conversely, some clades, e.g. *Eurotiales*, have much fewer N/Q-rich proteins, and in some cases likely lose them *en masse*, perhaps due to greater amyloid intolerance, although they contain relatively more non-N/Q-rich predicted prions. We find that recent mutational tendencies arising during *Saccharomycetes* evolution (i.e., increased numbers of N residues and a tendency to form more poly-N tracts), contributed to the expansion/development of the prion phenomenon. Variation in these mutational tendencies in *Saccharomycetes* is correlated with the population sizes of prion-like proteins, thus implying that selection pressures on N/Q-rich protein sequences against amyloidogenesis are not generally maintained in budding yeasts.

**Conclusions:**

These results help to delineate further the limits and origins of N/Q-rich prions, and provide insight as a case study of the evolution of compositionally-defined protein domains.

**Electronic supplementary material:**

The online version of this article (doi:10.1186/s12862-016-0594-3) contains supplementary material, which is available to authorized users.

## Background

Yeast prions are propagating alternative states of proteins. These states can be transmitted sustainably in the yeast *Saccharomyces cerevisiae* during budding, mating or laboratory infection protocols. The first well-characterized yeast prions, that underlie the [PSI+] and [URE3] prion states, are propagating amyloids (i.e., fibrillar beta-sheet aggregates) of the proteins Sup35p and Ure2p. The protein Sup35p is part of the translation termination complex. Formation of [PSI+] prions reduces the efficiency of translation termination and increases levels of nonsense-codon read-through [1, 2]. Such read-through has been shown to be a potential mechanism to uncover cryptic genetic variation [3, 4]. [URE3] causes upregulation of poor nitrogen source usage, even when rich sources are available [5–7]. Prion variants may be considered as diseases of *S. cerevisiae* in some contexts [8, 9]. A more recently discovered example, the [MOT3+] prion, has been shown to govern acquisition of multicellularity in *S. cerevisiae* [10]. There are now at least 10 known prions of *S. cerevisiae* that are propagated by amyloids [11, 12].

A common compositional feature of almost all amyloid-based yeast prions is bias for asparagine (N) and/or glutamine (Q) residues [11, 12]. A majority of them are N-rich (6/10 at the time of this analysis), rather than Q-rich. Bioinformatic surveys have revealed the existence of hundreds of proteins with such N/Q-richness in *S. cerevisiae* and diverse other fungi [13–15]. Evolutionary analysis showed that the [PSI+] prion N/Q bias is conserved across fungal clades that diverged >1 billion years ago, with only eight other *S. cerevisiae* proteins showing similar, phylogenetically deep patterns of N/Q bias conservation [14]. The [URE3] prion domain is unique to *Saccharomycetes*, with different parts of the domain demonstrating purifying selection (i.e., significant conservation of amino-acid identity from examination of codon position mutation rates), and variation in N/Q bias between clades [14, 16].

The peculiar composition of known prions has been exploited to computationally detect likely prions that were then tested experimentally for prion-forming ability [17]. Tests for in vitro and in vivo amyloid formation were combined with a Sup35 prion assay, wherein predicted prion-forming domains were fused to the C-terminal part of the Sup35p protein, and these constructs were then tested for the ability to produce [PSI+]-like states in yeast cells [17]. About twenty novel ‘prionogenic’ proteins were identified. The results from this survey have been used to train other algorithms to predict prion domains bioinformatically [18] (PrionW, PLAAC [19, 20]). On a related note, ‘scrambled’ forms of the Ure2p and Sup35p prion domains, that maintain the same amino-acid composition, can form prions in *S. cerevisiae*, indicating that prion formation is primarily determined by composition but not by specific sequence features [21, 22]. Building on these analyses, an amino-acid propensity scale for prion formation was developed and incorporated into the PAPA method for prion prediction [23, 24].

Putative prion domains from other *Saccharomycetes* (but not from fungal clades outside of this one) can make prions in *S. cerevisiae* or in their own cells, although this ability is sporadic [25–30], and can rely on small changes in the protein sequence [29]. Conversely, the full-length non-yeast protein CPEB from the sea hare *Aplysia californica* can form prions in *S. cerevisiae*, albeit with much less efficiency than native prions [31, 32]. Mutational experiments indicate that many N/Q-rich domains in *S. cerevisiae* may only be a small number of sequence mutations away from prion-forming ability, implying that natural selection may only act to keep aggregation propensities sufficiently low [33]; this may be an under-appreciated effect in the analysis of mammalian prion disease mutations [34, 35].

Several human proteins have prion-like N/Q-rich domains that have been directly linked to neurodegenerative diseases. Cytoplasmic aggregates of the RNA-binding protein FUS, which contains a Q-rich domain, are implicated in amyotrophic lateral sclerosis, and its aggregation has been re-capitulated in an induced *S. cerevisiae* proteinopathy [36]. Mutations in two yeast-prion-like proteins hnRNPA2B1 and hnRNPA1 initiate neurodegenerative disease in humans through amyloid formation [37]. HNRPDL has a yeast-prion-like domain, and has been linked to development of limb-girdle muscular dystrophy 1G [38]. Also, pathogenic proteins in at least nine other neurodegenerative disorders have disease-linked poly-Q expansions. Thus, the degree of conservation and variation of yeast prion domains has implications not just in fungi, but for human diseases as well.

Here, we probe how prion and prion-like proteins have evolved across the fungal kingdom. We discover that the ancestors of N-rich prion formers emerged during *Saccharomycetes* speciation, in tandem with a general dramatic increase in the number of N-rich proteins. Conversely, more ancient prion biases are Q-rich, at least back to the last common ancestor of fungi. Some fungal clades have very few N/Q-rich proteins, and in some cases likely lose them *en masse*. We find evidence that recent emergence of large populations of N/Q-rich proteins in *Saccharomycetes* may be partly due to mutational tendencies leading to more frequent initiation and elongation of poly-N runs. Variation in these mutational tendencies in *Saccharomycetes* is correlated with the population sizes of prion-like proteins, thus implying that selection pressure on N/Q-rich protein sequences to prevent their formation of amyloids is not generally maintained across *Saccharomycetes*.

## Methods

### Fungal proteomes

We downloaded complete proteomes of 169 fungi from various sources as listed in Additional file 1: Table S1.

### Evolutionary analysis

A phylogeny of fungi was obtained from NCBI Taxonomy (http://www.ncbi.nlm.nih.gov). Organismal phylogenetic trees were drawn using phyloT (http://phylot.biobyte.de) to generate a Newick format file, which was then input into Phylodendron (http://iubio.bio.indiana.edu/treeapp/treeprint-form.html). Orthologs for all of the *S. cerevisiae* proteins in all of the other fungi were calculated using the bi-directional best hits method. Families of paralogous proteins were determined using the CDHIT program (http://cd-hit.org). Removing the small numbers of putative paralogous duplications (identified using the CDHIT program) for N/Q-rich proteins has no effect on these observed trends for reported in this paper.

### Prion and prionogenic proteins

Prion and prionogenic sequence sets for *S. cerevisiae* were taken from the PrionHome database [11, 12]. Here, we analyze as groups: (i) the set of known prions, and (ii) a larger set made from these known prions plus other ‘prionogenic’ amyloid-forming proteins (PAFs). We initially included the non-N/Q-rich prion protein Mod5p in our analysis, which underlies the [MOD+] prion state [39], to check whether it acquires N/Q-rich domains in other clades (Table 1); but discovered that it does not gain any. The PAFs set includes the prionogenic proteins from the analysis of Alberti, et al. [17] that have been shown to form prions, through a SUP35C prion assay in conjunction with evidence for in vivo amyloid formation by the full-length proteins from the other assays. The list of PAFs is as follows: (UniProt IDs and standard names, well-characterized ‘known’ prion proteins have an asterisk): P05453, Sup35p*; P07884, Mod5p*; P09547, Swi1p*; P14922, Cyc8p*; P23202, Ure2p*; P25367, Rnq1p*; P32432, Sfp1p*; Q08972, New1p*; P54785, Mot3p*; Q02629, NUP100p*; P32588, Pub1p*; P40070, Lsm4p; P14907, Nsp1p; P18494, Gln3p; P32770, Nrp1p; P38180, YBL081W; P38216, YBR016W; P38429, Sap30p; P38691, Ksp1p; P40356, Pgd1p; P53894, Cbk1p; Q05166, Asm4p; Q08925, Mrn1p; Q12139, YPR022C; Q12221, Puf2p; Q12224, Rlm1p; Q12361, Gpr1p. Further evidence for the importance/relevance of many of the PAF proteins to the prion phenomenon and other aggregation-dependent phenomena in yeast is continuing to accumulate. For example, Lsm4 amyloids can both act as a [PSI+] prion inducer and prion clearer (the latter when overexpressed) [40–43], and can underlie the aggregation of P-bodies [44]. Also, fragments of Sap30p and Gpr1p have been shown to act as prion inducers [45]. The N/Q-rich regions of Nsp1p are important in mediating nucleoporin hydrogel formation, and interact in trans with the Sup35p prion domain [46].

**Table 1:** Summary of conservation of *S. cerevisiae* prion and PAF proteins in taxonomically defined sets of species*

Conservation level**	Has ortholog	Has ortholog and N/Q-rich domain	Has ortholog and PLAAC prediction	Has ortholog and PAPA prediction	Has ortholog and PrionW prediction
Known Prions					
within Saccharomycetes	11/11	10	10	10	8
within Ascomycota beyond Saccharomycetes	9	6	6	5	2
beyond Ascomycota	9	6	6	5	2
Total set of PAFs					
within Saccharomycetes	27/27	26	26	26	21
within Ascomycota beyond Saccharomycetes	20**	15	12	11	3
beyond Ascomycota	18**	13	11	9	3

*This table shows the fraction of the lists of prions and PAFs that are conserved in the taxonomic groups studied in this work. To be conserved ‘within Saccharomycetes’, a *S. cerevisiae* prion or PAF has to be found in any other *Saccharomycetes* species. To be conserved ‘within Ascomycota beyond Saccharomycetes’, a prion/PAF has to be found in any other Ascomycota species beyond the Saccharomycetes, and likewise for ‘beyond Ascomycota’. The columns denote the different cases simply an ortholog of the prion/PAF is detected, then an ortholog with an N/Q-rich domain, then an ortholog with a prion domain predicted by the PLAAC, PAPA or PrionW algorithms. This table includes the non-N/Q-rich prion former Mod5p

**One classed as within Saccharomycetes only, has horizontal transfer (HT) to non-Saccharomycetes detected in the evolutionary tree. The prion-like domain in this cases post-dates the HT

### Prion-like proteins

Prion-like proteins are defined in two ways: (a) through identification of compositional bias for N and Q residues, and (b) through application of algorithms designed to predict prion-forming domains.

N/Q-rich Proteins (NQPs) have N/Q-rich domains in them. For *S. cerevisiae* and all other fungal proteomes, N/Q-rich domains were determined using the LPS compositional-bias binomial probability minimization algorithm (with a maximum binomial P-value threshold of 1×10^−10^, derived from analyzing known prion-determinant domains [12, 14, 15, 47–49]), but testing three different criteria for expected amino-acid composition: *(i)* using the amino-acid composition for structured protein domains for each proteome (determined using blastp search against the ASTRAL database [50] with e-value threshold <1e–04 [51]); *(ii)* using equal expected amino-acid composition (i.e. =0.05), and *(iii)* using the average amino-acid composition of all the proteomes examined. Results are reported for criterion *(ii)*, but the same dominant trend for large-scale emergence of N-rich proteins in *Saccharomycetes* is observed regardless of the criterion used.

Also, we applied the PAPA and PLAAC prion prediction programs [19, 23, 24] to all the complete proteomes. The PAPA algorithm using an experimentally derived prion propensity score combined with explicit consideration of the intrinsic disorder. For PAPA, the default threshold for prion prediction was used. PLAAC uses a Hidden Markov Model trained on the composition of known prion-forming domains, which all have a pronounced bias for N and/or Q residues, and were all known or predicted intrinsically-disordered domains. For PLAAC, we used as a threshold the lowest COREscore value for the known prion-forming proteins (20.58 for Sfp1p). Also, the PrionW webserver [20] was applied to the PAF data set and their orthologs. Intrinsic disorder was annotated using IUPRED [52].

## Results and discussion

Firstly, we examine the evolutionary ancestry of the prions and other prionogenic amyloid-forming (PAF) protein sequences across the fungal kingdom. Then, we describe a dramatic large-scale emergence of N-rich prion-like proteins in the budding yeasts (*Saccharomycetes*), and how this contrasts with the notable lacks/losses of prion-like proteins in particular fungal clades/species. We show how the N-rich protein emergence in *Saccharomycetes* species is a striking trend that sets them apart from other fungi. We analyze how this trend is linked to recent mutational tendencies in this clade, and discuss the implications of our observations for prion formation.

**Fig. 1:**
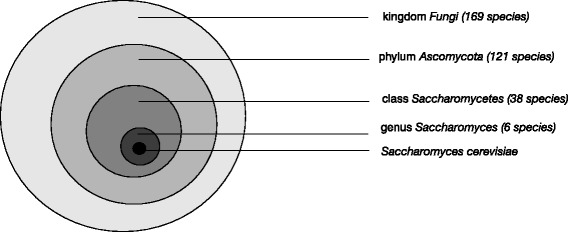
The taxonomic levels considered for orthologs of prions and PAFs. The number of species is given for each level

In general, we examine trends at three evolutionary depths: (i) within the class of budding yeasts *Saccharomycetes* (also known as *Hemiascomycetes*), (ii) within the phylum of the sac fungi *Ascomycota* but beyond the *Saccharomycetes*, and (iii) outside of *Ascomycota* in other fungi (Fig. 1). We examine the conservation of N/Q-richness and predicted prion status (annotated as described in Methods).

### Evolutionary origins of the ancestral sequences of prions and other prionogenic amyloid formers

**Fig. 2:**
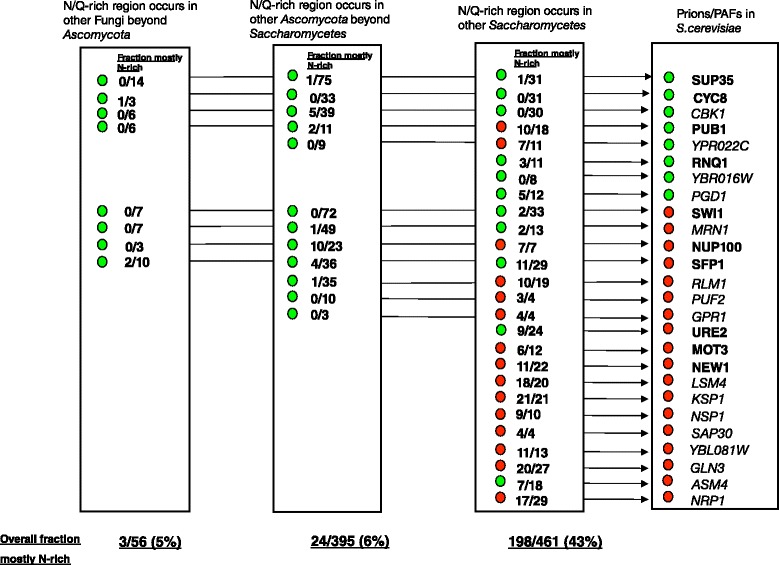
Summary of trends observed for the evolution of prions and other PAFs. The evolution of each prion/PAF is summarized. They are listed far right with prion gene names in bold, other PAFs in italics. Q-rich prions/PAFs are labeled with a green dot, N-rich with a red dot. Moving from right to left, we move deeper into the evolutionary past to a more ancient last common ancestor, and wider to more divergent clades of the fungi kingdom. First, we consider conservation in other *Saccharomycetes*, then in other *Ascomycota* beyond the *Saccharomycetes*, then finally in other Fungi beyond the *Ascomycota*. The fraction of orthologs with N/Q-rich domains in each of these groupings that are designated N-rich is listed. Where this is >0.5 the dot is red, otherwise it is green. At the bottom of these three columns is listed the overall fraction of N-rich

We find conspicuously distinct evolutionary ancestry for prions/PAFs if we separate them into N-rich and Q-rich cases. They are designated N-rich if they have smaller *P*-value from the LPS algorithm for N bias than for Q bias, and vice versa for Q-rich cases (see Methods for details). N-rich sequences dominate the set of prions/PAFs (18/26 cases), with almost all of these arising evolutionarily within the *Saccharomycetes* (Fig. 2). If these N-rich prions/PAFs arose earlier in fungal evolution, they had a Q-rich sequence which subsequently became N-rich within *Saccharomycetes* (Fig. 2). All but one of the N-rich prion/PAF domains arose within an evolutionarily short time frame, after the last common ancestor of *Saccharomycetes*, and before the whole genome duplication (WGD) event that occurred within *Saccharomycetes*. (A small minority (5/26) of the PAFs have ohnologs (i.e. WGD gene duplications), of which four maintain N/Q-rich predicted prion status.) The ancestor of the prion protein Rnq1p appeared in the same evolutionary epoch. Rnq1p is one of three mostly Q-rich prions that arose within *Saccharomycetes*, whose [PIN+]/[RNQ+] prion is required for the induction in wild strains of the [PSI+] prion made from Q-rich Sup35p [40, 53].

All *originally* Q-rich prion/PAF sequences arose before the last common ancestor of the *Saccharomycetes*, either within *Ascomycota*, or further back in fungal evolution (Fig. 2). We can see that ancestral Q-rich composition, back beyond the last common ancestor of the *Saccharomycetes,* occurs for 6/10 of the N/Q-rich prions (bold names in Fig. 2) and 12/26 of the PAFs overall (names in bold or italics, Fig. 2). For such Q-rich sequences of ancient origin, switching to N bias arises only rarely outside of *Saccharomycetes* yeasts (in 5-6 % of all orthologs) (Fig. 2). A particularly notable case is the Q-rich prion protein CYC8, which is a part of a global transcription repressor complex that controls the expression of ~7 % of yeast genes [54]. It is deeply conserved as a prion-like domain across diverse *Ascomycota* and in a few other fungal clades. Almost all of the putative prion-like domains for orthologs of CYC8 are Q-rich (66/67, i.e. with a single N-rich case). Such deep conservation of Q-rich prion-like domains (to before the last common ancestor of *Ascomycota*) for the prion proteins Sup35p, Cyc8p, Swi1p and others may be linked to a function other than prion formation. The prion domains of Sup35p and Ure2p have been shown to also have non-prion-forming functions [55, 56]. However, Pub1/Tia1 functions in stress granule assembly in mammals through aggregation mediated by its prion-like domain, a phenomenon that also arises in single-celled yeast [57, 58]. It is particularly intriguing that the Q-richness of such prion sequences has been deeply conserved across diverse fungal clades, since it has been shown through mutation experiments that Q-richness tends to lead to formation of toxic non-amyloid conformations in *S. cerevisiae*, whereas N-richness tends to produce benign propagating amyloids [59]. The chromatin remodelling factor Swi1p has distinct N-rich and Q-rich domains [60]. The N-rich domain, which has arisen recently in the evolution of *S. cerevisiae* and closely related yeasts, is required for the formation of the [SWI+] prion, which causes a partial loss of function phenotype [61, 62]; the deeply conserved Q-rich domain can modify aggregation patterns [60]. The N-rich and Q-rich regions function in causing a gain in sensitivity to Na+/Li + ions [60].

The conservation of the prion-like character of prions/PAFs for the three studied taxonomic groups is summarized in Table 1. For most prions/PAFs with orthologs ‘beyond Ascomycota’, or ‘within Ascomycota beyond Saccharomycetes’, prion-like domains are observed, either as N/Q-rich annotations or algorithmic prion predictions (Table 1). There is substantial agreement between prion/PAF ortholog annotations for N/Q-rich domains and algorithmic prion predictions (discussed in Additional file 2: Text S1).

### Emergence of N-rich proteins in Saccharomycetes yeasts

**Fig. 3:**
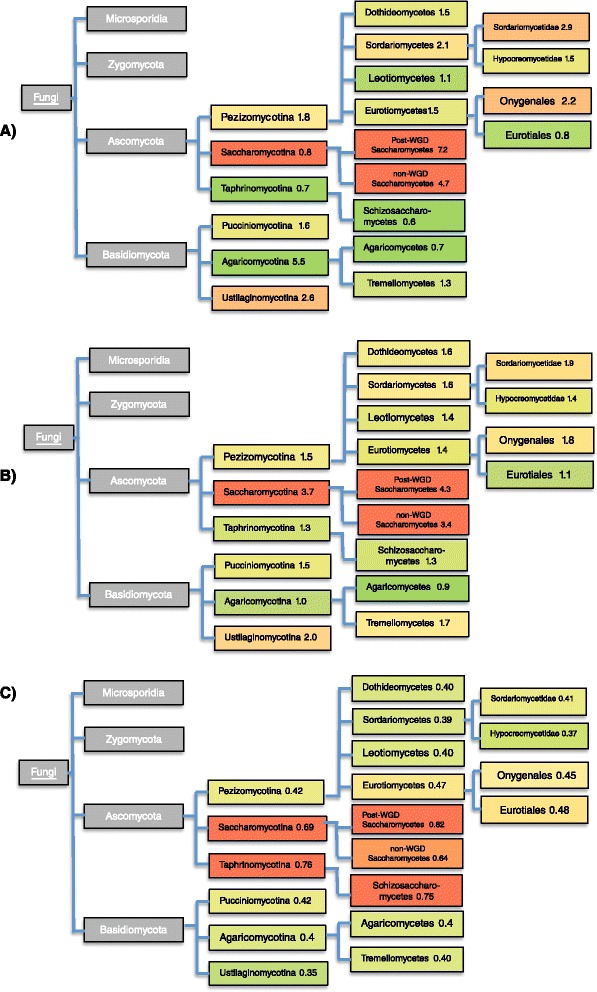
Overall trend in occurrence of prion-like proteins. **a** Summary of the trend presented in detail in Additional file 3: Figure S1 for the numbers of N/Q-rich proteins. The heatmap colour coding is the same as in that figure. The leaf nodes of this schematic tree as for the *Ascomycota* and *Basidiomycota* ‘trend clades’ from Additional file 3: Figure S1, i.e. the clades into which the tree can be split according to the obvious trends within these clades. The overall percentages are listed after the clade names. **b** Same plot as (A), except it is for the union of all of the prion predictions by the PLAAC and PAPA programs. Heatmap colour coding according to the numbers in column 2 of Additional file 7: Figure S3 is used. **c** Same plot as (A), except it is summarizing the trend presented in detail in Additional file 7: Figure S3 for total numbers of non-N/Q-rich prion predictions. To define non-N/Q-rich prion predictions, we used a strict threshold for N/Q bias (*P* = 1×10^−5^). The heatmap colour coding is the same as for Additional file 7: Figure S3

Is there any link between patterns of emergence of prion ancestors and prion-like proteins across the fungal kingdom? To answer this, we annotated all N/Q-rich proteins across the whole proteomes of the >160 fungal species under study. In doing this, we discovered a dramatic expansion in the number of N/Q-rich proteins in the *Saccharomycetes* clade, with all other clades having on average substantially fewer (Additional file 3: Figure S1 in detail, with a schematic summary in Fig. 3a). This is due to emergence of large numbers of N-rich prion-like domains, during *Saccharomyetes* evolution (Additional file 4: Figure S2). The trend for the evolutionary emergence of N-rich domains in prions and other PAFs is thus linked to a more general large-scale trend during *Saccharomycetes* evolution. This evolutionary trend for N/Q-rich domain genesis is replicated for numbers of prion predictions by the PAPA and PLAAC programs (Fig. 3b). N/Q-rich domains which arose within *Saccharomycetes* have significant functional linkage to transcription regulation, as determined by analysis of Gene Ontology process category enrichments (Additional file 5: Table S2, corrected *P*-values <0.05). N-rich prion-like domains (for example the one in Swi1) thus may have a specific functional influence on the recent evolutionary dynamics of transcriptional regulation pathways in the *Saccharomyces* genus. The Gene Ontology category enrichments (amongst many others) are also observed for the N/Q-rich proteins that occur beyond *Saccharomycetes* (with corrected P-values ≤1e–26). At least seven of the known prions/PAFs likewise function in regulation of transcription (the prions MOT3 [10], SWI1 [61], CYC8 [54], SFP1 [63] and the PAFs GLN3 [64], PGD1 [65] and RLM1 [66]). N-bias is not the only bias to become prominent in *Saccharomycetes*; there is also an emergence of more D-, E- and K-rich proteins (Additional file 6: Table S3, discussed in more detail below).

### Many fungal clades and species have few prion-like proteins and in some cases have likely lost them in their recent evolution

Some clades have very few N/Q-rich proteins or predicted prions and thus fewer possible N/Q-rich prions (Additional file 3: Figure S1 and Fig. 3). These include the *Eurotiales* (containing the filamentous fungi genus *Aspergillus*), the fission yeasts *Schizosaccharomycetes* and the *Agaricomycetes* (the class including the mushrooms)*.* Also, species in these clades contain few orthologs of the known prions/PAFs (Table 2). The dearth of likely prion-forming and prion-like proteins may be perhaps due to some mechanistic intolerance to their aggregation/propagation. Indeed, they may be too easily propagated to daughter cells in some fungal species, and thus subject to greater selection pressure on their sequences against formation of prion-forming domains. For *Eurotiales*, the most parsimonious explanation is that N/Q-rich domains and possible prions have been lost since their last common ancestor with other *Ascomycota* relatives.

**Table 2:** Clades (and species within *Saccharomycetes*) containing notably low percentages of prion and prion-like proteins

	% NQPs in proteome*	% prion predictions in proteome**	Fraction of known prions conserved as N/Q-rich	Fraction of known prions conserved as predicted prions**	Fraction of N/Q-rich PAFs conserved as N/Q-rich	Fraction of N/Q-rich PAFs conserved as predicted prions**
Clades						
*Eurotiales*	0.85 %	1.11 %	2.3/10	2.4/10	3.1/26 (12 %)	3.9/26 (15 %)
*Schizosaccharomycetes*	0.72 %	1.29 %	1.5/10	1.3/10	1.5/26 (6 %)	1.5/26 (6 %)
*Agaricomycetes*	0.69 %	0.94 %	0.8/10	0.6/10	0.8/26 (3 %)	1.3/26 (5 %)
Species within Saccharomycetes						
*Ogataea parapolymorpha (non-WGD species)*	1.12 %	1.61 %	3/10	2/10	4/26 (15 %)	4/26 (15 %)
*Ashbya gossypii (non-WGD species)*	1.70 %	1.51 %	5/10	3/10	8/26 (31 %)	4/26 (15 %)
For comparison:						
*Post-WGD Saccharomycetes*	7.19 %	4.34 %	7.5/10	6.8/10	16.6/26 (70 %)	15.8/26 (61 %)
*Non-WGD Saccharomycetes*	4.70 %	3.38 %	5.3/10	5.1/10	10.2/26 (32 %)	9.5/26 (37 %)

*For clades, overall average values

**The union of prion predictions for the PLAAC and PAPA programs is used

Notably, two of the clades with fewest overall N/Q-rich proteins or prion predictions (*Eurotiales* and *Schizosaccharomycetes*) have some of the largest numbers of non-N/Q-rich prion predictions (Additional file 7: Figure S3 and Fig. 3c; a strict N/Q bias threshold of 1×10^−5^ is used). Although these are speculative predictions, this may indicate that these clades harbour differently composed cohorts of functional amyloid-forming proteins.

Substantial losses of prion-like proteins also occur in two individual *Saccharomycetes* species (Additional file 3: Figure S1). These are the non-WGD species *Ogataea parapolymorpha* and *Ashbya gossypii.* The species *O. parapolymorpha* has the lowest level of conservation of known prions and PAFs in *Saccharomycetes* (Table 2). *O. parapolymorpha* is an atypical thermotolerant yeast with a relatively high GC% (percentage guanidine + cytidine) genome that can grow on methanol, acquiring large numbers of cellular peroxisomes in the process; it also has an unusual thermotolerance mechanism linked to production of trehalose, a sugar normally found in insects. The filamentous yeast *A. gossypii* has undergone substantial genome evolution since divergence from its close relative *E. cymbalariae*, gaining higher GC% and losing transposons and 10 % of its genome size [67]. The high GC% of these two genomes (51 % for *A. gossypii* and 48 % for *O. parapolymorpha;* the highest and third-highest GC% values of the *Saccharomycetes* species examined) may be a contributing factor to the loss of N-rich prion-like domains in particular: N codons are one sixth guanidine/cytidine (whereas Q codons are half). Correlation with variation in GC% is discussed in more detail below. *O. parapolymorpha* and *A. gossypii* share three conserved prion proteins (Cyc8p, Sup35p and Sfp1p). Sup35p and Sfp1p are functionally linked prions that exert control over translation accuracy [63]. This is thus evidence for selection to maintain a small core of prions, despite many others being lost.

### A distinctive trend for formation of prion-like proteins in Saccharomycetes yeasts

**Fig. 4:**
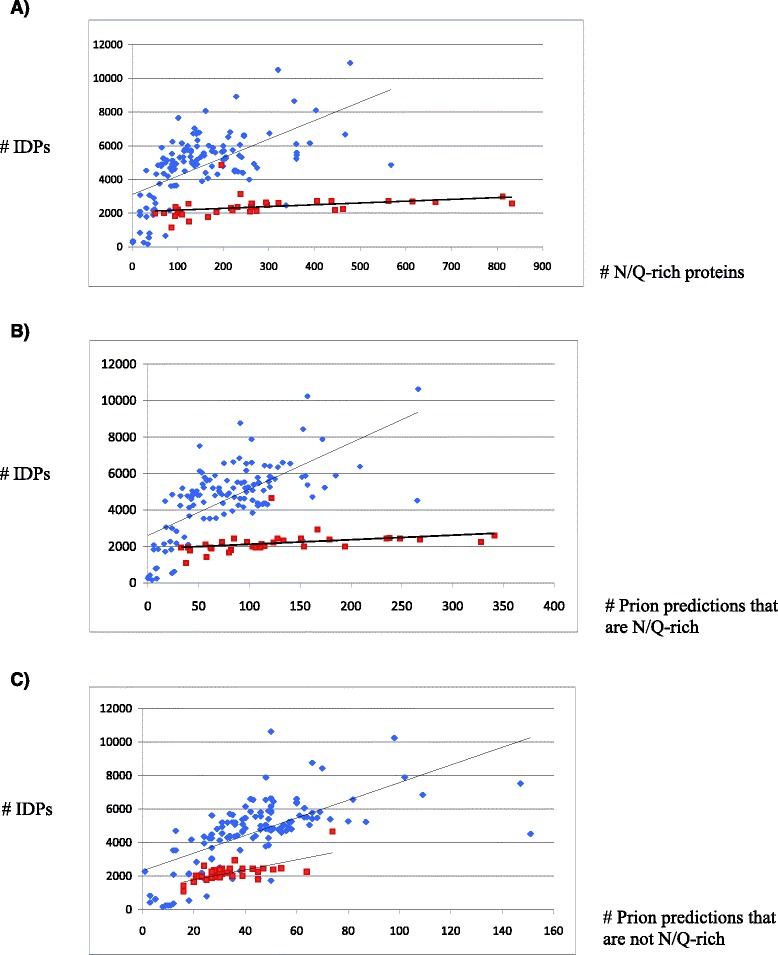
Numbers of intrinsically disordered proteins (IDPs) versus numbers of N/Q-rich proteins or prion predictions. **a** Plot of number of IDPs versus numbers of N/Q-rich proteins. Proteins with ID regions >30 residues were counted as IDPs. We only consider IDPs that do not have N/Q-rich domains in the IDP totals. Saccharomycetes species are red points, and non-Saccharomycetes blue. The trend line for both is shown. The Pearson correlation coefficients are: *R* = 0.135 (*P* = 0.03) Saccharomycetes, *R* = 0.358 (*P* < 1e–07) non-Saccharomycetes. **b** Same as (A), but with IDPs versus predicted prion proteins (the union of PAPA and PLAAC predictions for each proteome) that are N/Q-rich. *R* = 0.139 (*P* = 0.03) Saccharomycetes, *R* = 0.444 (*P* < 1e–07) non-Saccharomycetes. The IDP totals are for those that have no prion predictions in them (by either PAPA or PLAAC), i.e. all of the proteins with prion predictions are removed. **c** Same as (A), but for the subset of predicted prions that are not N/Q-rich. *R* = 0.49 (*P* < 1e–07) Saccharomycetes, *R* = 0.422 (*P* < 1e–07) non-Saccharomycetes. To define non-N/Q-rich prion predictions, we use a strict threshold for N/Q bias (*P* = 1×10^−5^). As above in part (B), the IDP totals are for those that have no prion predictions in them

Since prion and prion-like proteins are intrinsically disordered, we surmised that maybe the trends in variation of N/Q-rich proteins or predicted prions are due to a more general trend for variation in the number of intrinsically-disordered proteins (IDPs) across fungal evolution. Thus, we compared the numbers of IDPs with the numbers of N/Q-rich proteins and prion predictions, for each proteome (Fig. 4). In general, we find some degree of correlation between numbers of IDPs and prion-like proteins. This may be because many intrinsically disordered regions (including those that contain N/Q-rich regions) are evolving neutrally or nearly neutrally, with little negative selection pressure. Also, such intrinsically disordered regions (including those that contain N/Q-rich regions) may have some organizational function in the cell that makes their precise amino-acid composition unimportant. In Fig. 4, we find a distinct trend for *Saccharomycetes* yeasts that sets them apart from non-*Saccharomycetes. Saccharomycetes* occupy the lowest portion of the scatter plots with a shallow correlation, where they segregate from all the other fungi. The highest numbers of N/Q-rich proteins (400+, or 150+ for those also predicted as prions by the PAPA and PLAAC programs), are in the genera *Candida* and *Tetrapisispora*, and in the species *N. dairensis* and *L. elongisporus*. This is the case whether we consider compositionally-defined N/Q-rich proteins, or PAPA/PLAAC-predicted prions that are N/Q-rich (Fig. 4a-b). The shallow distinct correlation for *Saccharomycetes* implies that additional N/Q-rich domains tend to arise by mutation without formation of many additional IDPs, and this is at a rate that sets them apart from other fungi. Notably, considering only non-N/Q-rich algorithmic prion predictions makes the trend for *Saccharomycetes* less distinct, implying that the trend observed is primarily due to N/Q-richness, and that numbers of non-N/Q-rich prion predictions are more correlated with numbers of IDPs generally (Fig. 4c). Algorithmically predicted non-N/Q-rich yeast prions are a largely untested cohort, and their exact design principles have yet to be determined.

Motivated by this distinct trend for *Saccharomycetes* yeasts, and given that N/Q-rich proteins (NQPs) are compositionally defined, we checked whether their numbers in fungal proteomes are correlated with other compositional characteristics (Table 3). We defined two special types of N and Q composition, % ‘lone’ N and Q and % ‘run’ N and Q. ‘Lone’ N and Q do not occur in homopeptide runs and are surrounded on either side by ≥2 non-N/Q residues. ‘Run’ N and Q occur in runs of 3–5 residues. Both ‘lone’ and ‘run’ N and Q residues are counted only from proteins that are not N/Q-biased (using a strict P-value threshold of <1×10^−5^), and that are not predicted to be prions by PAPA or PLAAC. Overall in fungi, and in other fungal clades examined for comparison, we find significant correlations for NQP numbers arising out of Q percentages (Table 3). However, when we specifically examine the *Saccharomycetes* clade, we see a different situation. There is a prominent correlation for % of run N, with other compositional traits having less significant or non-significant correlations (Table 3). These results indicate that the sizes of populations of N/Q-rich proteins in *Saccharomycetes* yeasts is directly linked to a mutational tendencies for more N residues, particularly in poly-N runs. Lower %GC (% guanidine + cytidine in the DNA) may lead to a higher proportion of Ns for initiation of runs (since N codons are AT-rich/GC-poor). For *Saccharomycetes*, we see also an increase in K-, D- and E-rich proteins, and a depletion of A-, G-, P- and R-rich proteins compared to other fungal clades (Additional file 6: Table S3). Notably, K residues are encoded by the most AT-rich set of codons in the genetic code, while A, G, P and R comprise the amino-acid residues encoded by GC-rich codons. Also, in line with this observation, we see that within *Saccharomycetes*, GC% has high positive correlation with the occurrence of alanine-rich proteins, and high negative correlation with the occurrence of isoleucine- and lysine-rich proteins (Additional file 8: Table S4). These latter two amino acids are encoded by AT-rich codons. Thus, GC% is an important contributor to the occurrence of compositionally biased regions in *Saccharomycetes* proteins, including N/Q-rich regions.

**Table 3:** Comparison of correlations of percentage NQPs versus other compositional features of fungal proteomes*

	%GC	% lone NQ †	% run NQ †	% lone N †	% lone Q †	% run N †	% run Q †
Trend clades**	−0.14 (NS)	0.22 (NS)	0.84 (0.00016)	0.18 (NS)	−0.11 (NS)	0.41 (NS)	0.70 (0.0037)
Saccharomycetes	−0.68 (0.00014)	0.42 (0.0054)	0.73 (<0.00001)	0.51 (0.0007)	−0.39 (0.009)	0.74 (<0.00001)	0.21 (NS)
Onygenales	0.54 (NS)	0.03 (NS)	0.02 (NS)	−0.09 (NS)	0.07 (NS)	−0.11 (NS)	0.15 (NS)
Eurotiales	0.27 (NS)	−0.33 (NS)	−0.21 (NS)	−0.37 (NS)	0.20 (NS)	−0.33 (NS)	−0.04 (NS)
Eurotiomycetes ***	0.16 (NS)	−0.24 (NS)	0.30 (NS)	0.20 (NS)	0.60 (0.0005)	0.22 (NS)	0.37 (0.03)

*Spearman rank correlation coefficients (with P-values) for one-tailed test, for the trend clades; Pearson correlation coefficients for the other analysis. The two most significant for each row are in bold

**The trend clades are the obvious groupings from examining the trend across Ascomycota and Basidiomycota for %NQPs in Additional file 3: Figure S1, and depicted in schematic Fig. 3. No significant results are found by simply considering the six subphyla (three from Ascomycota, three from Basidiomycota)

***Contains both the Onygenales and Eurotiomycetes and a small number of other organisms

**†**‘Lone’ N and Q do not occur in homopeptide runs and are surrounded on either side by ≥2 non-N/Q residues. ‘Run’ N and Q occur in runs of 3–5 residues. Both ‘lone’ and ‘run’ N and Q residues are counted only from proteins that are non-N/Q-biased proteins (using a strict P-value threshold of <1×10^−5^), and that are not predicted to be prions by PAPA or PLAAC. ‘Lone NQ’ and ‘run NQ’ are simply the sums of ‘lone N’ and ‘lone Q’, and ‘run N’ and ‘run Q’ respectively

However, %GC is not correlated with numbers of NQPs across the fungal kingdom (Table 3). Indeed, there are several notable clades that have similar %GC but drastically different % NQPs. For example, *Schizosaccharomyces* and *Saccharomyces* species have similar %GC (~37 % versus ~39 %), but *Schizosaccharomyces* have much fewer NQPs and prion predictions (Fig. 3 and Additional file 3: Figure S1). A similar situation arises in the *Basidiomycota*, where *Ustilaginomycotina* have much more NQPs and prion predictions than *Agaricomycotina*, but have similar %GC (~54 % versus ~52 %). Thus, the precise nature of the selection pressures that contribute to the populations of NQPs remains to be elucidated fully. It has been shown that part of the Ure2p prion-forming domain is under purifying selection in *Saccharomycetes*, whereas another part of the domain varies widely in its N and Q composition [14]. Whether in some sequences this variation is partly caused by diversifying or positive selection (i.e., significantly increased amino-acid mutation rate from examination of codon position mutation rates) will require further developments in molecular evolution models for biased sequences.

## Conclusions

The evolutionary vista for the ancestors of prion and prion-like proteins changed substantially in *Saccharomycetes* budding yeasts. During *Saccharomycetes* evolution, large-scale formation of N-rich regions occurred. This thus may have provided a trigger for the expansion and development of the prion phenomenon and so consequently were born the ancestral sequences of the prions Ure2p, Mot3p and New1p, and other N-rich PAF proteins of *S. cerevisiae*. Thus, new prion domains could have initially arisen from the formation of sufficiently long poly-N/Q tracts (particularly poly-N tracts) [68]. Certain individual newly-formed N-rich domains subsequently have been maintained to perform a function that may or may not be related to prion formation [14]. Other factors being conducive, these evolutionarily novel N-rich domains could evolve to produce benign propagating amyloids in *S. cerevisiae* [59]. Also within the same epoch (before the whole genome duplication in budding yeasts), the Rnq1p protein required for [PSI+] induction has arisen as a novel protein. Variation in recent mutational tendencies for more N residues, particularly in the form of poly-N tracts, is correlated with population sizes of N/Q-rich proteins in individual *Saccharomycetes* yeast species. Given the correlation that we see between numbers of N/Q-rich proteins and numbers of short poly-N tracts in other proteins, these results suggest that there is no clade-wide maintenance of selection pressure on N/Q-rich protein sequences to prevent N/Q-rich protein aggregation. This may be either because in many species there is cellular machinery to prevent/handle them effectively, or because they do not often enough tend to aggregate. In the amoeba *Dictyostelium*, there are large numbers of N/Q-rich proteins, and experiments on Sup35p aggregation indicate that there are cellular mechanisms preventing their aggregation generally [69]. Such mechanisms may also allow larger populations of N/Q-rich proteins in the *Tetrapisispora* and *Candida* clades, and are of interest for the analysis of diseases in humans that are linked to prion-like proteins or poly-Q repeat expansions, such as Huntington’s disease. Also, the tendency to form poly-N homopeptide runs per se may be under selection variably in different lineages of budding yeasts, to control the evolution of functional N/Q-rich domains. Indeed, the relative selective burden on the protein sequences per se against harmful aggregation may vary as the potency of anti-aggregation cellular mechanisms varies. Assessment of these latter hypotheses would require experimental evolution investigations in tandem with novel theoretical developments. The evolution of mutation rates and the heterogeneity of rates for different types of mutation is a current area of interest in experimental evolution analysis [70, 71].

### Availability of supporting data

Proteomes analyzed can be downloaded from the links listed in Additional file 1: Table S1. All other data sets supporting the results of this article are included within the article (and its additional files).

## Additional files

Additional file 1: Table S1. List of fungal proteomes and their sources (weblinks). (XLS 37 kb) Additional file 2: Text S1. Comparison of annotations of N/Q-rich proteins and prion predictions by the PAPA, PLAAC and PrionW programs. (DOCX 163 kb) Additional file 3: Figure S1. Large phylogenetic tree showing the trend in numbers of prion-like N/Q-rich proteins (NQPs). Colour-coding is according to a heatmap with green for low N/Q-rich numbers and red for high. The heatmap scale is indicated in the figure. The numbers of N-, Q-, N/Q- and Q/N-rich regions are listed for each species. Q/N-rich are regions that have a mingled bias of Qs and Ns, but mostly Q; similarly, for N/Q-rich. Clades are labelled where they branch off in the tree. (PNG 737 kb) Additional file 4: Figure S2. Large phylogenetic tree for N-rich/Q-rich ratio. Colour-coding is according to a heatmap with green for low N-rich/Q-rich ratio and red for high. The heatmap scale is indicated in the figure. Listed for each species are the total number of N-, Q-, N/Q- and Q/N-rich proteins and the N-rich/Q-rich ratio, which is the number of N-rich divided by the number of Q-rich proteins. Q/N-rich are regions that have a mingled bias of Qs and Ns, but mostly Q; similarly, for N/Q-rich. (PNG 737 kb) Additional file 5: Table S2. Gene Ontology process category enrichments. (TXT 1 kb) Additional file 6: Table S3. Other biases, that become prominent or depleted in *Saccharomycetes*. (DOCX 17 kb) Additional file 7: Figure S3. Large phylogenetic tree showing numbers of PLAAC, PAPA and combined PLAAC and PAPA (union of the two sets), and non-N/Q-rich PLAAC/PAPA predictions. Colour-coding is according to a heatmap with green for low percentage of non-N/Q-rich prion predictions and red for high. The heatmap scale is indicated in the figure. For this tree, for counting non-N/Q-rich PLAAC/PAPA predictions we use a strict threshold for N/Q bias (*P* = 1×10^−5^). (PNG 790 kb) Additional file 8: Table S4. Further correlations referenced in the manuscript. (DOC 43 kb)

## Abbreviations

AT: adenine + thymidine
DNA: deoxyribonucleic acid
GC: guanidine + cytidine
IDP: intrinsically disordered protein
N/Q-rich: asparagine/glutamine-rich
NQP: N/Q-rich protein
PAF: prionogenic amyloid former
RNA: ribonucleic acid
WGD: whole genome duplication
