# Supplementary material for: Emergence and evolution of yeast prion and prion-like proteins
Source: BMC Evol Biol. 2016 Jan 25;16:24. doi: 10.1186/s12862-016-0594-3 (PMC4727409; doi:10.1186/s12862-016-0594-3)
Supplement: Additional file 2: Text S1. — Comparison of annotations of N/Q-rich proteins and prion predictions by the PAPA, PLAAC and PrionW programs. (DOCX 163 kb) [file 12862_2016_594_MOESM2_ESM.docx]

**Supplementary Text S1: Comparison of annotations of N/Q-rich proteins and prion predictions**

This survey gives us the opportunity to compare the predictions made by different prion prediction algorithms (PAPA and PLAAC, and the PrionW webserver (Toombs, et al. 2012; Ross, et al. 2013; Lancaster, et al. 2014; Zambrano, et al. 2015)), and to assess how these compare to N/Q-rich domain annotations. This is all the more interesting since the prions/PAFs were used to train or afterwards assess these algorithms. The substantial majority of prions predicted in prion/PAF orthologs by PAPA, have N/Q-rich domains (345/384, 90%) (see Figure below). Of the remainder, most have a mild N/Q bias (24/39, 62%), as judged by the LPS program (binomial P<1x10^–6^). Similarly, for PLAAC and PrionW, most prion domain predictions are N/Q-rich (744/899, 83% PLAAC; 123/30, 95% PrionW). Also for these two programs, most of the remainder have a mild N/Q bias (101/155, 65% PLAAC; 6/7, 86% PrionW). Also, the PAPA and PLAAC programs largely agree, with only 45/384 PAPA predictions (12%) not predicted by PLAAC. In contrast, PrionW agrees less with the other programs with 100/130 (77%) in common with PLAAC, and 88/130 (68%) predictions in common with PAPA. A small minority of N/Q-rich domains in orthologs are not predicted as prions by the algorithms applied (114/893, 13%). These results indicate that the prion prediction programs are largely dependent on detection of N/Q bias. PrionW, which includes looking for predicted amyloid-nucleation sequences yields a smaller, more refined list of predictions, and predicts very few prions outside the *Saccharomycetes*, favoring sequences in closely related species that are more like known prion-forming domains of *S. cerevisiae*.

**Figure: Venn diagram showing the general consensus between methods for prion prediction and N/Q-rich annotation.**

142

114

16

12

7

1

20

398

246

80

0

PLAAC

899

13

0

6

25

PrionW

130

NQ-rich

893

PAPA

384
