# Supplementary figures and images for: Emergence and evolution of yeast prion and prion-like proteins
Source: BMC Evol Biol. 2016 Jan 25;16:24. doi: 10.1186/s12862-016-0594-3 (PMC4727409; doi:10.1186/s12862-016-0594-3)

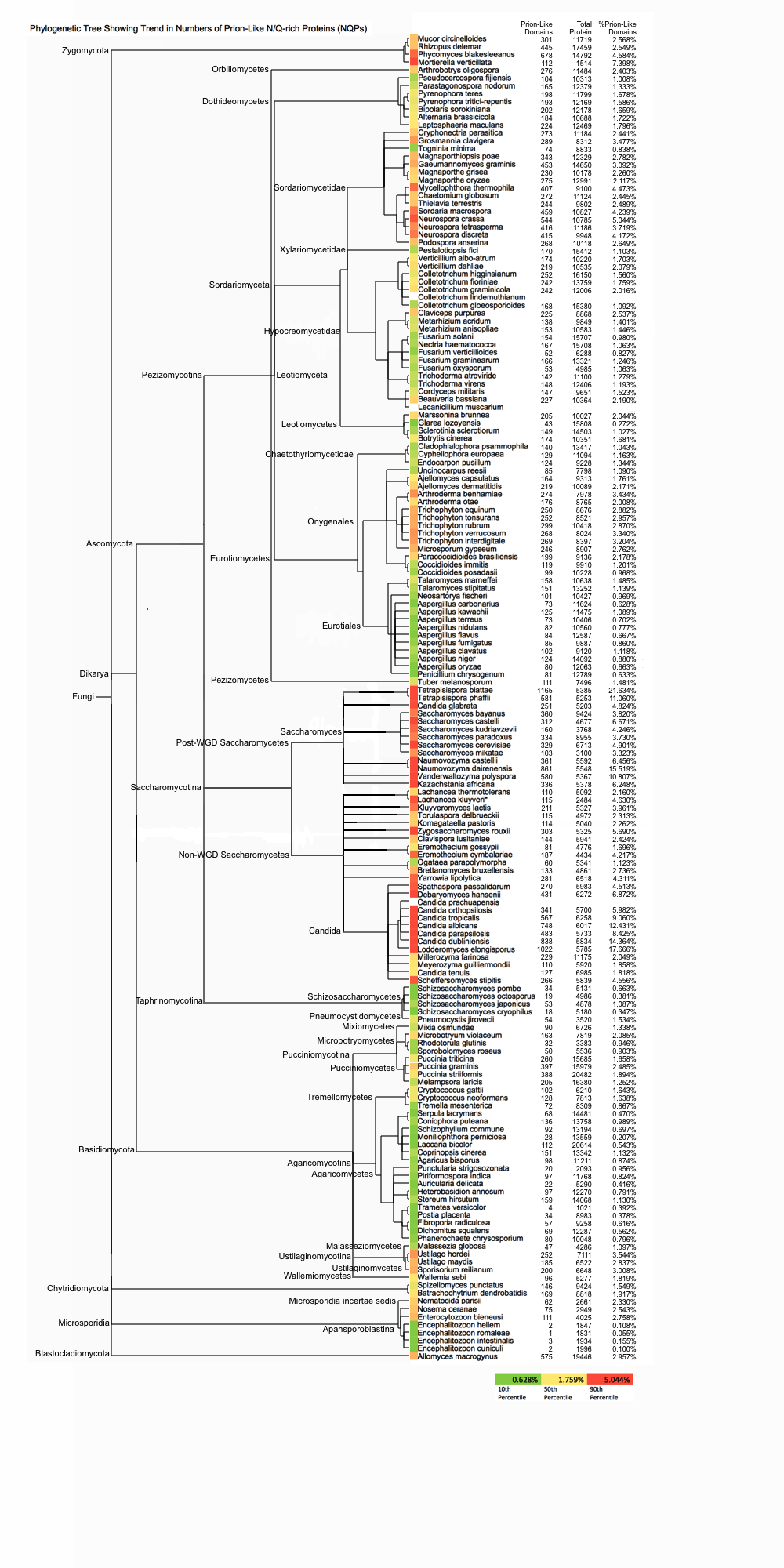

Supplement: Additional file 3: Figure S1. — Large phylogenetic tree showing the trend in numbers of prion-like N/Q-rich proteins (NQPs). Colour-coding is according to a heatmap with green for low N/Q-rich numbers and red for high. The heatmap scale is indicated in the figure. The numbers of N-, Q-, N/Q- and Q/N-rich regions are listed for each species. Q/N-rich are regions that have a mingled bias of Qs and Ns, but mostly Q; similarly, for N/Q-rich. Clades are labelled where they branch off in the tree. (PNG 737 kb) [file 12862_2016_594_MOESM3_ESM.png]

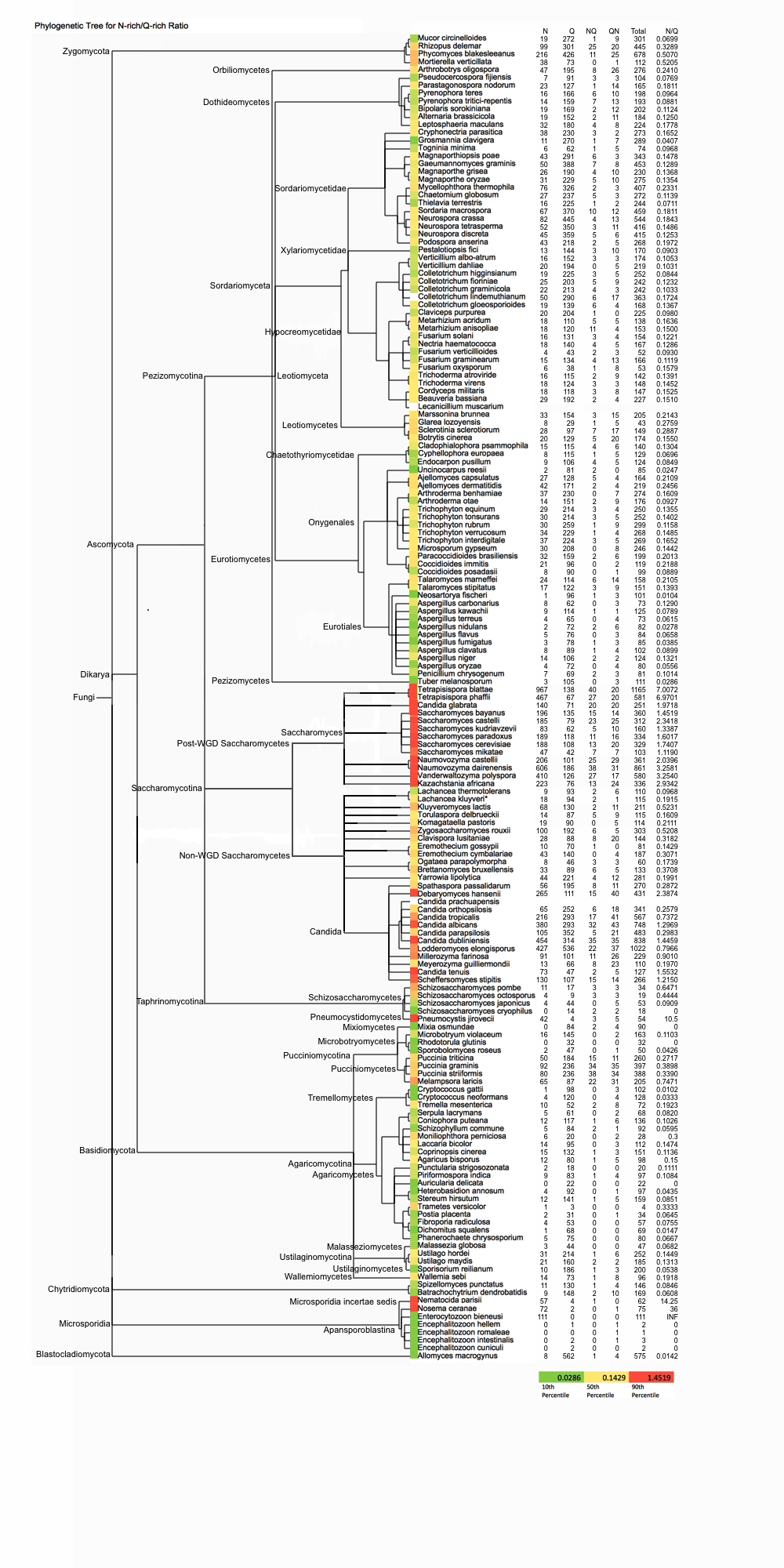

Supplement: Additional file 4: Figure S2. — Large phylogenetic tree for N-rich/Q-rich ratio. Colour-coding is according to a heatmap with green for low N-rich/Q-rich ratio and red for high. The heatmap scale is indicated in the figure. Listed for each species are the total number of N-, Q-, N/Q- and Q/N-rich proteins and the N-rich/Q-rich ratio, which is the number of N-rich divided by the number of Q-rich proteins. Q/N-rich are regions that have a mingled bias of Qs and Ns, but mostly Q; similarly, for N/Q-rich. (PNG 737 kb) [file 12862_2016_594_MOESM4_ESM.png]

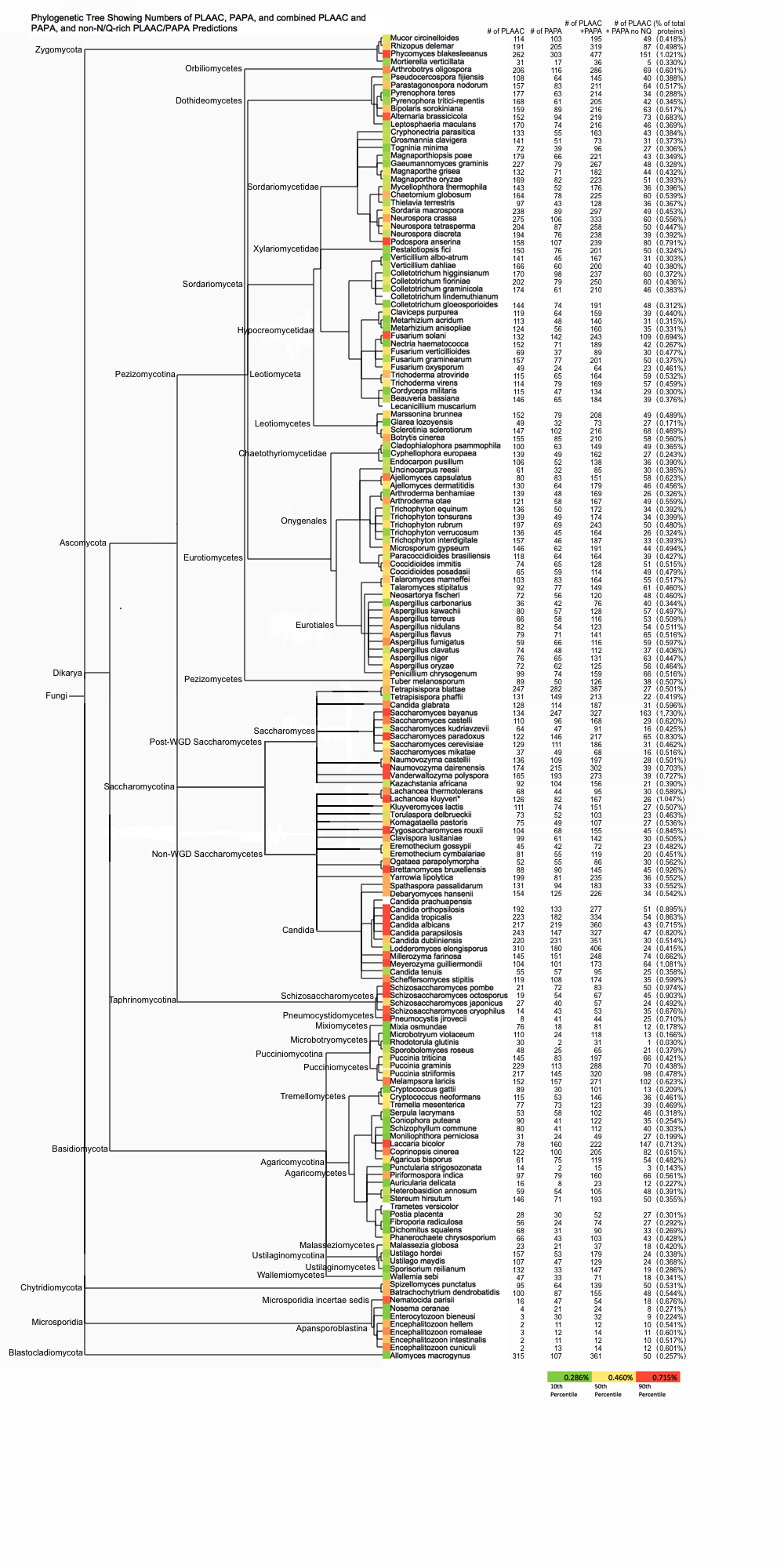

Supplement: Additional file 7: Figure S3. — Large phylogenetic tree showing numbers of PLAAC, PAPA and combined PLAAC and PAPA (union of the two sets), and non-N/Q-rich PLAAC/PAPA predictions. Colour-coding is according to a heatmap with green for low percentage of non-N/Q-rich prion predictions and red for high. The heatmap scale is indicated in the figure. For this tree, for counting non-N/Q-rich PLAAC/PAPA predictions we use a strict threshold for N/Q bias (P = 1×10^−5^). (PNG 790 kb) [file 12862_2016_594_MOESM7_ESM.png]
