# Supplementary material for: Emergence and evolution of yeast prion and prion-like proteins
Source: BMC Evol Biol. 2016 Jan 25;16:24. doi: 10.1186/s12862-016-0594-3 (PMC4727409; doi:10.1186/s12862-016-0594-3)
Supplement: Additional file 6: Table S3. — Other biases, that become prominent or depleted in Saccharomycetes. (DOCX 17 kb) [file 12862_2016_594_MOESM6_ESM.docx]

**Supplementary Table 3: Other biases that become prominent or depleted in *Saccharomycetes †***

| **Residue Type** | **Ranking of the *Saccharomycetes* in the clades and percentages of proteins with the biases** |
| --- | --- |
| ***P-value threshold = 1e–06*** | |
| *Become prominent 🡪* | |
| D (Aspartate) | 2* (8.2%), 3** (7.4%) |
| K (Lysine) | 1* (21.5%), 3** (14.7%) |
| E (Glutamate) | 2* (15.8%), 3** (15.1%) |
| *Become depleted 🡪* | |

| A (Alanine) | 11** (9.6%), 13* (6.3%) |
| --- | --- |

| G (Glycine) | 11** (6.6%), 12* (4.5%) |
| --- | --- |
| P (Proline) | 12** (4.5%), 13* (3.2%) |
| R (Arginine) | 12** (1.9%), 13* (1.4%) |
|  | |
| ***P-value threshold = 1e–10*** | |
| *Become prominent 🡪* | |
| D (Aspartate) | 2* (2.1%), 3** (2.1%) |
| K (Lysine) | 1* (5.4%), 3** (3.4%) |
| E (Glutamate) | 2* (5.2%), 3** (5.1%) |
| *Become depleted 🡪* | |
| A (Alanine) | 11** (2.4%), 13* (1.4%) |
| G (Glycine) | 11** (1.4%), 12* (0.8%) |
| P (Proline) | 12** (1.6%), 13* (1.0%) |
| R (Arginine) | 12** (2.8%), 13* (1.7%) |
|  | |
| ***P-value threshold = 1e–14*** | |
| *Become prominent 🡪* | |
| D (Aspartate) | 2* (0.8%), 3** (0.8%) |
| K (Lysine) | 1* (1.7%), 3** (1.2%) |
| E (Glutamate) | 2* (2.7%), 3** (2.5%) |
| *Become depleted 🡪* | |
| A (Alanine) | 11** (1.0%), 13* (0.6%) |
| G (Glycine) | 11** (0.7%), 12* (0.4%) |
| P (Proline) | 11** (0.7%), 12* (0.4%) |
| R (Arginine) | 12** (0.3%), 13* (0.2%) |

* post-WGD (i.e. post- whole genome duplication). ** non-WGD.

† Biases for which the post-WGD and non-WGD saccharomycetes are consistently

in the top or bottom three of clades (out of a total of 13), when ranked in decreasing order of % for the clades

examined, as detailed in Figure 2.
