# Supplementary material for: Emergence and evolution of yeast prion and prion-like proteins
Source: BMC Evol Biol. 2016 Jan 25;16:24. doi: 10.1186/s12862-016-0594-3 (PMC4727409; doi:10.1186/s12862-016-0594-3)
Supplement: Additional file 8: Table S4. — Further correlations referenced in the manuscript. (DOC 43 kb) [file 12862_2016_594_MOESM8_ESM.doc]

**Supplementary Table 4: Further correlations***

| **GC% vs % lone N or Q** | |
| --- | --- |
| **Trend clades**** |  |
| %-lone-N | -0.93 (<0.00001) |
| %-lone-Q | 0.72 (0.006) |
| %-run-N | -0.80 (0.001) |
| %-run-Q | 0.51 (NS) |
| **Saccharomycetes** |  |
| %-lone-N | -0.90 (<0.00001) |
| %-lone-Q | 0.68 (0.0004) |
| %-run-N | -0.93 (<0.00001) |
| %-run-Q | 0.21 (NS) |
|  | |
| **GC% vs percentage biased proteins in Saccharomycetes proteomes***** | |
| %A-rich-proteins | 0.81 (<0.00001)  (all GC-rich codons) |
| %R-rich-proteins | 0.61 (0.002) |
| %V-rich-proteins | 0.30 (NS) |
| %P-rich-proteins | 0.29 (NS) |
| %G-rich-proteins | 0.16 (NS) |
| %L-rich-proteins | -0.03 (NS) |
| %F-rich-proteins | -0.30 (NS) |
| %H-rich-proteins | -0.32 (NS) |
| %E-rich-proteins | -0.36 (NS) |
| %S-rich-proteins | -0.40 (NS) |
| %D-rich-proteins | -0.44 (0.038) |
| %T-rich-proteins | -0.57 (0.005) |
| %I-rich-proteins | -0.74 (0.00006)  (all AT-rich codons) |
| %K-rich-proteins | -0.84 (<0.00001)  (all AT-rich codons) |

* Spearman rank correlation coefficients with P-values for one-tailed test in brackets, for the trend clades; Pearson correlation coefficients for the other analysis.

** The trend clades are the obvious groupings from examining the trend across Ascomycota and Basidiomycota for %NQPs in Suppl. Figure 2, and depicted in schematic Figure 2. No significant results are found by simply considering the six subphyla (three from Ascomycota, three from Basidiomycota).

*** Same threshold for the LPS algorithm as for N/Q-rich proteins is used here (P<=1e-10). Correlations for residues for which there are very few biased proteins (<10 in all species), are not listed.
